# Supplementary material for: Does the size of rewards influence performance in cognitively demanding tasks?
Source: PLoS One. 2020 Oct 21;15(10):e0240291. doi: 10.1371/journal.pone.0240291 (PMC7577432; doi:10.1371/journal.pone.0240291)
Supplement: S1 Appendix — (DOCX) [file pone.0240291.s001.docx]

**S1 Appendix: Pre-registration**

**Have any data been collected for this study already?**

No

**What's the main question being asked or hypothesis being tested in this study?**

*Hypothesis 1*

A larger piece-rate reward will lead to better performance.

*Hypothesis 2*

A larger piece-rate reward will lead to worse performance.

**Describe the key dependent variable(s) specifying how they will be measured.**

*Performance*

Performance is the sum of the number of solved tasks in the adding exercise and the anagram exercise.

**How many and which conditions will participants be assigned to?**

Two. One group will get 30 Danish crowns regardless of performance. Furthermore, they will get 2 Danish crowns for each solved task. The other group will get 30 Danish crowns regardless of performance. Furthermore, they will get 10 Danish crowns per solved task. No participants will receive less than 40 Danish crowns total.

**Specify exactly which analyses you will conduct to examine the main question/hypothesis.**

I will test the model:

$$total number of solved tasks=\beta_{0}+\beta_{1}*large reward+\beta_{2}*female+\beta_{3}*conscientiousness +u$$

by OLS using robust standard errors (also known as standard errors with Eicker, Huber, White or a hyphenation of these names as the prefix). I will use a significance level of 5 %.

**Any secondary analyses?**

Yes. I will add a questionnaire in order to shed light on the underlying theoretical mechanisms. This analysis is dependent on the result from the main analysis.

*If a large piece-rate reward has a statistically significant, negative effect on performance*

Hypothesis 1: The group with a large piece-rate reward was less focused on the tasks.

Hypothesis 2: The group with a large piece-rate reward was less intrinsically motivated.

This will be tested with a t-test for difference in means using Welch’s t-test. A Bonferroni correction of the significance level will be used. Since two tests are carried out, the significance level becomes 2.5 %.

*If a large piece-rate reward has a statistically significant, positive effect on performance*

Hypothesis 1: The group with a large piece-rate reward exerted a larger effort.

This will be tested with a t-test for difference in means using Welch’s t-test. Correction of the significance level is unnecessary since only a single test will be carried out. Thus, a significance level of 5 % will be used.

*If a large piece-rate reward does not have a statistically significant effect on performance.*

Hypothesis 1: The group with a large piece-rate reward was less focused on the tasks.

Hypothesis 2: The group with a large piece-rate reward was less intrinsically motivated.

Hypothesis 3: The group with a large piece-rate reward exerted a larger effort.

This will be tested with a t-test for difference in means using Welch’s t-test. A Bonferroni correction of the significance level will be used. Since three tests are carried out, the significance level becomes 1.67 %.

Furthermore, I will have descriptive statistics for my variables. This will be done for both groups together as well as each group separately.

**How many observations will be collected or what will determine sample size? No need to justify decision, but be precise about exactly how the number will be determined.**

I will try to have 102 participants. However, I have not used this laboratory and this participant pool before so I might end up with fewer participants than this. At the time of the writing of the Danish version of the preregistration, 54 persons had signed up for the experiment. The participants can reject participating on the day of the experiment or choose to not show up. Furthermore, they will be removed from the analysis if they fail to follow the instructions.

**Anything else you would like to pre-register? (e.g., data exclusions, variables collected for exploratory purposes, unusual analyses planned?)**

The different variables used in the analyses described above are defined as written below.

*Large reward*

Large reward is a dummy variable which takes the value 1 if the participant has been in the group with the large piece-rate reward and the value 0 if the participant has been in the group with the small piece-rate reward.

*Female*

This variable takes the value 1 if the participant is female and the value 0 if the participant is male.

*Conscientiousness*

The sum of the answers to questions 3 and 8 (R[eversed]) from TIPI.[1]

*Effort*

The sum of answers to questions 1, 3 (R), 5 (R), and 6 from the last questionnaire. [1]

*Intrinsic motivation*

The sum of answers to questions 2, 4 (R), 7 (R), and 8 from the last questionnaire.[1]

*Focus*

The sum of answers to questions 9, 10 and 11 (R) from the last questionnaire.[2]

Furthermore, I will calculate Cronbach’s α for the questions about effort, intrinsic motivation and focus.

[1] Note that while we pre-registered the use of a sum, we have relied on the average since this is the correct procedure.

[2] Note that while we pre-registered the use of a sum, we relied on an average in order to facilitate comparison across variables.
