# Supplementary material for: Does the size of rewards influence performance in cognitively demanding tasks?
Source: PLoS One. 2020 Oct 21;15(10):e0240291. doi: 10.1371/journal.pone.0240291 (PMC7577432; doi:10.1371/journal.pone.0240291)
Supplement: S2 Appendix — (DOCX) [file pone.0240291.s002.docx]

**S2 Appendix**

Answers to these questions are not related to your reward. For each of the following statements, please indicate how true it is for you, using the following scale:

| 1 | 2 | 3 | 4 | 5 | 6 | 7 |
| --- | --- | --- | --- | --- | --- | --- |
| Not at all true |  |  | Somewhat true |  |  | Very true |

| **Statement** | **Your indication** |
| --- | --- |
| I tried very hard on these tasks. |  |
| I thought these tasks were quite enjoyable. |  |
| I didn’t put much energy into this. |  |
| I thought these tasks were boring. |  |
| I didn’t try very hard to do well at these tasks. |  |
| It was important to me to do well at these tasks. |  |
| These tasks did not hold my attention at all. |  |
| These tasks were fun to do. |  |
| I thought of the reward while doing the tasks. |  |
| Thinking of the reward distracted me during the task solving. |  |
| During task solving, I was able to remain fully focused on solving the tasks. |  |
