# Supplementary material for: Does the size of rewards influence performance in cognitively demanding tasks?
Source: PLoS One. 2020 Oct 21;15(10):e0240291. doi: 10.1371/journal.pone.0240291 (PMC7577432; doi:10.1371/journal.pone.0240291)
Supplement: S3 Appendix — (DOCX) [file pone.0240291.s003.docx]

**S3 Appendix**

Conscientiousness is constructed from the following two questions:

*I see myself as:*

*[…]*

*3: Dependable, self-disciplined.*

*[…]*

*8: Disorganized, careless.*

The participants choose a number from the following Likert scale:

*1 = Disagree strongly*

*2 = Disagree moderately*

*3 = Disagree a little*

*4 = Neither agree nor disagree*

*5 = Agree a little*

*6 = Agree moderately*

*7 = Agree strongly*

The answer to 8 is then reversed (meaning 7 is taken as 1, 6 as 2 etc.), and the average of these two questions is then a participant’s score for conscientiousness (not that the sum, as per the pre-registration). Cronbach’s α has not been calculated for the TIPI questions as this a validated questionnaire to which we have made no changes. Furthermore, the TIPI has been shown to be valid despite having a low alpha-value (Gosling, et al., 2003). Gosling et al. (2003) show evidence that the correlations between results from TIPI and a BFI-test are between 0.65 and 0.87. For conscientiousness the correlation is 0.75.
