# Supplementary material for: Does the size of rewards influence performance in cognitively demanding tasks?
Source: PLoS One. 2020 Oct 21;15(10):e0240291. doi: 10.1371/journal.pone.0240291 (PMC7577432; doi:10.1371/journal.pone.0240291)
Supplement: S4 Appendix — (DOCX) [file pone.0240291.s004.docx]

**S4 Appendix**


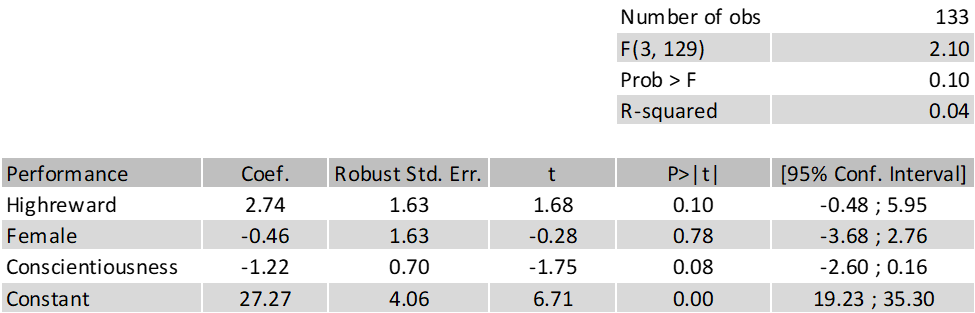


Figure 4A: Result from main regression excluding data from the first two sessions

|  | *Focus* | *Intrinsic motivation* | *Effort* |
| --- | --- | --- | --- |
| t Stat | -1.16 | 0.51 | 1.74 |
| P(T<=t) two-tail | 0.25 | 0.61 | 0.08 |
| 98.33 % confidence interval (low-high) | -0.83 ; 0.29 | -0.40 ; 0.61 | -0.11 ; 0.70 |
| Cronbach's α | 0.57 | 0.76 | 0.73 |

Figure 6A: t-tests for equality of means excluding data from the first two sessions

|  | Effort | | Intrinsic motivation | | Focus | |
| --- | --- | --- | --- | --- | --- | --- |
|  | 1 | 2 | 3 | 4 | 5 | 6 |
| Mediator | 2.28***  (0.85) | 2.60***  (0.90) | 1.78**  (0.76) | 1.69**  (0.74) | 0.44  (0.63) | 0.39  (0.65) |
|  |  |  |  |  |  |  |
| Gender and conscientiousness included | NO | YES | NO | YES | NO | YES |
| Constant included | YES | YES | YES | YES | YES | YES |
| Number of obs | 131 | 131 | 131 | 131 | 131 | 131 |
| R-squared | 0.05 | 0.09 | 0.05 | 0.07 | <0.01 | 0.03 |

Figure 7A: Relationship between performance and mediators excluding data from the first two sessions. *, **, and *** mean the variable is statistically significant at the 10 %, 5 %, or 1 % level.


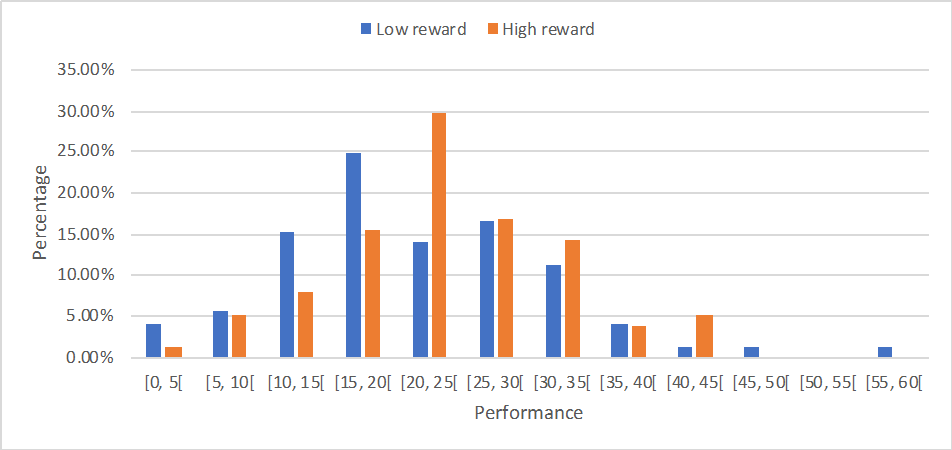


Figure 8: Distribution of performance in each condition.

Fig 9: Descriptive statistics, divided in to first and second round of data collection. Numbers in parentheses are standard deviations.
